# The Downregulation of Both Giant HERCs, *HERC1* and *HERC2*, Is an Unambiguous Feature of Chronic Myeloid Leukemia, and HERC1 Levels Are Associated with Leukemic Cell Differentiation

**DOI:** 10.3390/jcm11020324

**Published:** 2022-01-10

**Authors:** Muhammad Shahzad Ali, Stefano Magnati, Cristina Panuzzo, Daniela Cilloni, Giuseppe Saglio, Barbara Pergolizzi, Enrico Bracco

**Affiliations:** 1 Department of Clinical and Biological Science, Medical School, University of Torino, 10043 Orbassano, Italy; muhammadshahzad.ali@unito.it (M.S.A.); stefano.magnati886@edu.unito.it (S.M.); cristina.panuzzo@unito.it (C.P.); giuseppe.saglio@unito.it (G.S.); 2 Department of Oncology, Medical School, University of Torino, 10043 Orbassano, Italy; enrico.bracco@unito.it

**Keywords:** ubiquitin system, large HERCs, TKIs, CML, differentiation

## Abstract

Large HERC E3 ubiquitin ligase family members, *HERC1* and *HERC2*, are staggeringly complex proteins that can intervene in a wide range of biological processes, such as cell proliferation, DNA repair, neurodevelopment, and inflammation. Therefore, mutations or dysregulation of large *HERCs* is associated with neurological disorders, DNA repair defects, and cancer. Though their role in solid tumors started to be investigated some years ago, our knowledge about *HERCs* in non-solid neoplasm is greatly lagging behind. Chronic Myeloid Leukemia (CML) is a model onco-hematological disorder because of its unique and unambiguous relation between genotype and phenotype due to a single genetic alteration. In the present study, we ascertained that the presence of the *BCR-ABL* fusion gene was inversely associated with the expression of the *HERC1* and *HERC2* genes. Upon the achievement of remission, both *HERC1* and *HERC2* mRNAs raised again to levels comparable to those of the healthy donors. Additionally, our survey unveiled that their gene expression is sensitive to different Tyrosine Kinases Inhibitors (TKIs) in a time-dependent fashion. Interestingly, for the first time, we also observed a differential HERC1 expression when the leukemic cell lines were induced to differentiate towards different lineages revealing that HERC1 protein expression is associated with the differentiation process in a lineage-specific manner. Taken together, our findings suggest that HERC1 might act as a novel potential player in blood cell differentiation. Overall, we believe that our results are beneficial to initiate exploring the role/s of large HERCs in non-solid neoplasms.

## 1. Introduction

To some extent, Chronic Myeloid Leukemia (CML) can be considered a model disease not only in the onco-hematology field but more broadly in oncology. It is a clonal myeloproliferative disorder characterized by a single genetic alteration affecting the hematopoietic stem cells. Indeed, the balanced reciprocal translocation t(9;22) (q34;q11) gives rise to a constitutively active tyrosine kinase oncoprotein, BCR-ABL, that is the causative and functional lesion of CML. Thus, in CML, there is an unambiguous relation between genotype and phenotype [1]. Ultimately, BCR-ABL would lead to a progressive block of differentiation and increased genetic instability [2].

The CML can be effectively treated by selectively targeting BCR-ABL with small molecules tyrosine kinase inhibitors (TKIs), which by inhibiting the kinase activity, drive the cells towards apoptosis. The first tyrosine kinase inhibitor (TKI) developed and successfully used in the clinic was Imatinib [3,4]. Though most CML patients responded very well to Imatinib therapy, a minority relapsed [5]. This is due to several reasons ranging from acquired BCR-ABL tyrosine kinase point mutations to overexpression of multidrug resistance gene (*MDR1*) [6] or, alternatively, to the overexpression of Src kinases family members [7,8,9,10]. To overcome these hindrances, novel TKIs have been developed and introduced in the clinic, including Nilotinib and dual-specificity second-generation Src and BCR-ABL kinase inhibitors, such as Bosutinib, Dasatinib, and the more potent Ponatinib [11]. They show faster responses, lower rates of short-term disease progression, and deeper overall molecular response rates [5]. Due to its broader tyrosine kinase spectrum, Dasatinib has been proven to successfully inhibit the key signaling players involved in the pathogenesis of CML development. When compared to Imatinib, Dasatinib displays hematologic and non-hematologic associated toxicity effects that partially limit its usage [10]. Hence, alternative ways to identify new therapeutic targets/strategies are still required to drive deeper molecular responses, enabling more patients to attempt TKI discontinuation. 

The E3 ubiquitin ligases regulate several biological processes through timely ubiquitination and degradation of many cellular proteins responsible for receptor endocytosis, intracellular trafficking, signal transduction, DNA damage recognition and repair, differentiation, and apoptosis [12,13,14]. Consistently, the malfunctioning of E3 ligases leads to the development of numerous human diseases [15], such as central nervous system disorders, inflammation, metabolic dysfunctions, and cancers [16,17], including hematologic malignancies [18]. Several ubiquitin ligases are mutated, overexpressed, or deleted in hematologic malignancies contributing to the pathogenesis of the diseases through accumulation, or excessive degradation, of their substrates which perform critical functions that regulate hematopoietic cell growth and differentiation [15,18,19]. Recently, it has been reported by our group that the *HERC1* gene expression dysregulation and some myeloid-related disorders and the physical interaction between BCR-ABL1 and HERC1 [20]. HERC1, together with HERC2, are giant E3 ubiquitin ligases with a molecular weight exceeding 500 kDa. Both have a catalytic HECT domain at their carboxyl terminus that contains a conserved cysteine residue essential for the catalytic activity [15,17]. 

However, currently, specific inquiries aiming to assess the potential role of the giant HERCs in myeloid-related neoplasia have been minimal. Thanks to the efforts made by the Human Protein Atlas Project (proteinatlas.org), it has emerged that the differential gene expression of HERCs E3 ligase members might act as prognostic factors in a few solid tumors. 

In the current study, we investigated the *HERC1* and *HERC2* gene expression in two different cohorts of CML patients during the elapsed time between diagnosis and one year of TKIs (Imatinib and Dasatinib) treatment. Additionally, for the first time, the HERC1 gene and protein expression level changes were assessed when leukemic cell lines were induced to differentiate. These findings shed light on the large E3 ubiquitin ligase HERC1 as a novel potential and emerging player in blood cell differentiation.

## 2. Materials and Methods

### 2.1. Patient Samples and Cell Lines Culture

A total of 78 chronic myeloid leukemia were included in this study. Out of 78, 30 specimens (PB = 15 and BM = 15) were exclusively collected at diagnosis and 19 at remission for initial *HERC2* gene expression analysis. Furthermore, specimens were obtained, at different time points (0, 3, 6, and 12 months after the starting TKIs treatment), from 29 CML patients treated either with Imatinib (*n* = 15) or with Dasatinib (*n* = 14). We also included 16 healthy volunteers. All the samples were collected and processed in the Laboratory of Molecular Medicine and Oncology, Department of Clinical and Biological Sciences of the Hospital San Luigi Gonzaga Orbassano (TO), after written informed consent (local ethic committee S. Luigi Gonzaga Hospital 201/2014). The K562, HEL cell lines were cultured with RPMI-1640 and supplemented with heat-inactivated 10% fetal bovine serum for all cell lines (20% FBS for primary CML cells), 500 U/mL penicillin, and 0.5 mg/mL streptomycin. Cells were cultured at 37 °C in a humidified atmosphere flushed with 5% CO_2_.

### 2.2. Quantitative Real-Time PCR

Mononuclear cells from healthy volunteers and CML samples at each stage were separated on a Ficoll–Hypaque density gradient. Total RNA was extracted in TRIzol Reagent solution (Ambion, Waltham, MA USA), reverse transcribed, and then transcript quantification analysis was performed as described elsewhere [21]. For *HERC1*, *HERC2,* and the *GUSB* genes mRNA quantification, specific assays (assay IDs for Hs01032486_m1 for *HERC1*, Hs01117656_m1 for *HERC2* and *GUSB* Hs00939627_m1, and -Applied Biosystems, Thermo Fisher Scientific, Massachusetts, MA, USA) were used. The large *HERCs* Ct values obtained by RT-qPCR were normalized with respect to the Ct counterparts of *GUSB* and expressed as 2^−∆∆Ct^. A universal human references RNA (Stratagene, San Diego, CA, USA) was used to calibrate the assay.

### 2.3. CD34^+^, CD3^+^ and CD19^+^ Cells Separation

The isolation of CD34^+^, CD3^+^, and CD19^+^ cell fraction was achieved through the direct CD34 Progenitor, CD3, and CD19 Cell Isolation Kit (Miltenyi Biotec Inc., Auburn, CA, USA), following the manufacturer’s instructions. Briefly, 10^8^ cells were resuspended in 300 μL of ACD-Buffer (0.5% of bovine serum albumin (BSA) and 2 mM EDTA in PBS pH 7.2) and incubated with 100 μL of FcR Blocking Reagent and 100 μL of positive CD34 MicroBeads for 30 min at 4 °C. Magnetic separations of CD34^+^ fraction were performed by using the MACS separator with the appropriate MACS (MS) Column after its rehydration with an appropriate volume of ACD-buffer. Subsequently, the cell suspension was deposited on the column, filtered, and washed 3 times with ACD-buffer washes. Flow-through, containing unlabeled CD34 negative (CD34^−^) cells, were also collected. Positively labeled cells (CD34^+^), which remain magnetically linked to the column, were flushed out by firmly pushing the plunger into the column with an appropriate amount of buffer in a collection tube. The CD34^+^ cells were confirmed through FACS analysis. Similarly, the isolation of CD3^+^ and CD19^+^ cell fraction was achieved by resuspending MNC cells (10^8^) in 100 μL of ACD buffer and incubated for 15 min at 4 °C with 40 μL of CD3 or CD19 Microbeads. The subsequent magnetic separation was performed following the same protocol used to elute the bound CD34^+^ cells. 

### 2.4. Western Blot Analysis

Protein concentration was determined using the Bio-Rad DC Protein Assay (Bio-Rad). 100 µg of total protein were loaded and run onto 4–14% precast gradient gel (Criterion TM TGX Stain-Free TM) electrophoresis and transferred to PVDF (Bio-Rad, Hercules, California, CA, USA) membranes by using the protocol described elsewhere [22]. Blots were blocked in TBS (Tris-HCl pH7.6, 150 mM NaCl) plus 5% nonfat milk for 1 h at room temperature (RT) and then decorated with appropriate antibodies (HERC1 A301-904A, Bethyl Laboratories, Inc., Montgomery, TX, USA, Tubulin sc-23948, and p44/42 MAPK (ERK1/2) #4696s, phospho-p44/42 MAPK (T202/Y204) #4377s; Vinculin MA5-11690, Sigma-Aldrich; BCR Cell Signaling Tech, phospho-Tyr, sc-7020, Santa Cruz Biotechnology, Dallas, TX, USA; in PBS-Tween 0.5% overnight at 4 °C. Membranes were then washed with PBS-Tween 0.5% 3 times for 15 min each, incubated with appropriate peroxidase-linked secondary antibody (Santa Cruz Biotechnology) for 1 h at RT, and washed again in PBS-Tween 0.5%. Specific binding was detected using an enhanced chemiluminescence system (Clarity Western ECLSubstrate #170-5061, Bio-Rad, Hercules, CA, USA).

### 2.5. Induction of Differentiation and Flow Cytometry

To induce differentiation, exponentially grown K-562 and HEL cells (5 × 10^5^ cells/mL) were seeded in 10 mL of respective growth medium and exposed to K-562 with 250 nM PMA and 20 U of erythropoietin (EPO; Pfizer, Europe MA EEIG 1050 Bruxelles) separately and HEL with 250 nM PMA for 48 h [23,24]. Morphological changes of cells were observed using a phase-contrast microscope. Following treatment, cells were collected by centrifugation (500× *g* for 3 min), and each group was then washed in FACS buffer (PBS, 1 mM EDTA, 2% FBS, 0.1% sodium azide) and then resuspended in 500 μL blocking buffer (equal volumes of FACS buffer and FBS) for 10 min at room temperature. The cells were again washed with FACS buffer 2 times and incubated for 20–30 min in the dark with fluorescently conjugated antibodies PE Mouse Anti-Human CD61 (VI-PL2, BioLegend, San Diego, CA, USA). Subsequently, cells were washed twice with 1X PBS, resuspended in FACS buffer, and analyzed using a fluorescence-activated cell sorter (FACS) BD flow cytometer to detect the percentages of differentiated cells. Results were analyzed on FlowJo^®^ software version 10.5.3 (FlowJo, LLC, Ashland, OR, USA).

### 2.6. Immunofluorescence 

Cytospins were prepared using primary cells from CML patients, K562 (before and after treatment), and healthy donor cells. Immunofluorescence was performed as described [25]. Slides were analyzed by fluorescence confocal microscopy (LSM5110; Carl Zeiss MicroImaging, Inc., Jena, Germany); the images were captured using the 63X objective. The intensity of the signal from approximately 80 cells from each condition was evaluated through the use of ImageJ software. For each sample, repeated in triplicate, 3 independent measurements were performed, and each measurement was normalized on background noise. The mean intensity ± standard deviation δ (ds), expressed as fluorescence intensity/number of pixels was calculated and plotted using the program GraphPad Prism (GraphPad Software, San Diego, CA, USA).

### 2.7. Benzidine Staining 

Erythroid differentiation was evaluated by counting blue benzidine-positive cells (hemoglobin expressing cells) by benzidine staining described elsewhere [26]. Briefly, the EPO treated K-562 cells were suspended in a benzidine staining solution containing 0.2% benzidine (Sigma-Aldrich #B3503-5G) in 10% H_2_O_2_ (Sigma-Aldrich, Tokyo, Japan) and 0.5 M glacial acetic acid (Sigma-Aldrich). Following 2 min of staining, the differentiated cells were stained blue, and images were captured by phase-contrast microscopy. The percentage of benzidine-positive cells was the number of blue cells divided by the total number of cells.

### 2.8. Statistical and Correlation Analysis 

The large *HERC1* and *HERC2* gene expression values were first tested for normality by the Shapiro–Wilk test. Afterward, the values obtained at different time points from diagnosis to remission (3, 6, and 12 months) were analyzed and compared with non-parametric Kruskal–Wallis and Wilcoxon tests. The results were then corrected with a posthoc Bonferroni test. Eventually, correlation analysis coefficients (*ρ*) were determined using the Spearman test. Analyses were carried out by using the GraphPad Prism v5.0 (GraphPad Software, Inc., La Jolla, CA, USA) and R (https://cran.r-project.org/) software (R Core Team, Vienna, Austria). 

## 3. Results 

### 3.1. Baseline Features of the Inquired Cohorts

Baseline characteristics of healthy subjects and CML patients are summarized in Table 1. The present retrospective study was carried out on 29 CML patients under TKIs therapy, and all of them were positive for the *p210 BCR-ABL* rearrangement at diagnosis. Among them, 3 patients showed a co-expression of *b3a2/b2a2* fusion transcripts. The patients were either treated with Imatinib (*n* = 15) or Dasatinib (*n* = 14). The median age was 54 and 63.5 years for Imatinib and Dasatinib, respectively. The healthy donors (*n* = 16) with ages ranging between 21–63 years were included as the control. The genders were equally represented with the only exception of the patients’ cohort treated with Dasatinib, in which males are predominantly represented.

### 3.2. HERC2 Gene Expression Is Severely Down-Modulated in CML Specimens but Returns to Normal Levels upon Remission

**Figure 1:**
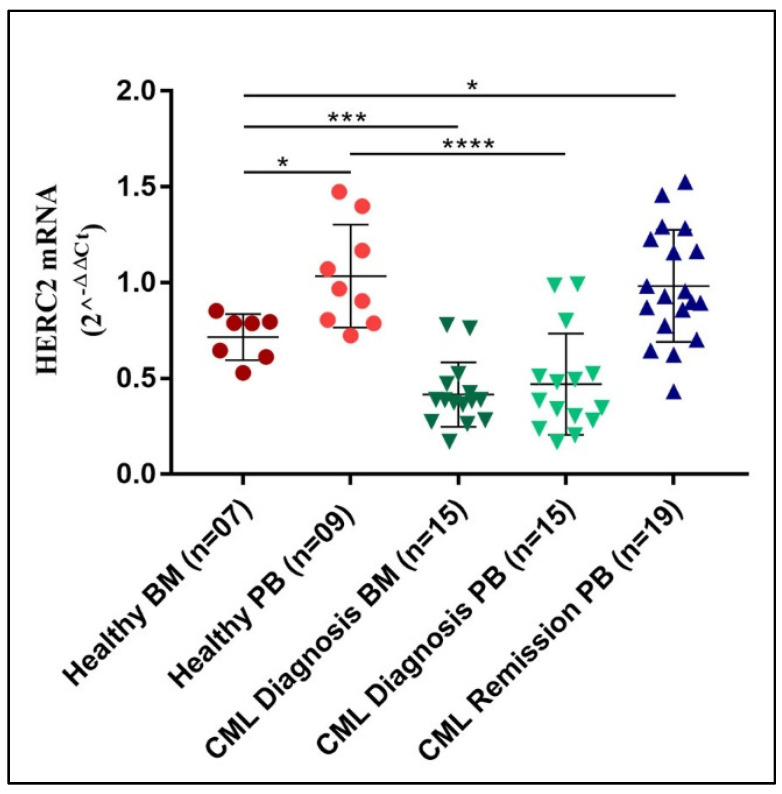
*HERC2* gene expression is differentially regulated PB and BM from healthy subjects, and it is down-modulated in newly diagnosed CML patients. *HERC2* gene was differentially expressed in BM and PB specimens from healthy subjects, displaying a significantly lower transcript amount in BM. When compared to the controls, newly diagnosed CML patients showed lower HERC2 mRNA levels, both in PB and BM samples. Remarkably, when CML patients reached remission, the HERC2 gene expression raised again to levels comparable to those encountered of healthy donors. *p* ≤ 0.05 = *; *p* ≤ 0.001 = ***; *p* ≤ 0.0001 = ****.

The assessment of the large *HERCs* transcripts’ amount in a few solid tumors reveals that they might act as prognostic markers (www.proteinatla.org). However, their role in non-solid tumors has started to be explored quite recently. In our previous study, we reported an association of *HERC1* gene expression with some myeloid-related disorders [20]. Curiously, besides our findings, the knowledge regarding the other large HERC sub-family member, *HERC2*, is currently missing in blood malignancies. Hence, we initially investigated the *HERC2* gene expression in a cohort of healthy subjects (*n* = 16) by assessing its mRNA abundance both in bone marrow (BM, *n* = 7) and peripheral blood (PB, *n* = 9) samples. Our analysis revealed that the *HERC2* mRNA level was slightly, but significantly (*p* < 0.05), different in PB and BM specimens with median values of 0.97 and 0.71, respectively (Figure 1). Then we assessed the *HERC2* gene expression in CML patients at diagnosis (*n* = 30) and once they reached complete cytogenetic remission (*n* = 19). Differently from what we observed in the healthy subjects, the *HERC2* transcript level in PB (*n* = 15) and BM (*n* = 15) samples collected at diagnosis were rather comparable, though, in terms of absolute values, the *HERC2* mRNA amount was significantly lower when compared to the mRNA amount of controls (Figure 1). Similar to what has been observed for *HERC1*, we did not detect any significant difference in terms of *HERC2* gene expression between the PB and BM samples from CML samples obtained at diagnosis. Eventually, once CML patients reached remission (*n* = 19), the *HERC2* transcript raised again to levels comparable to those of the healthy donors. On the whole, the *HERC2* gene expression is differentially regulated in healthy PB and in BM, while it is significantly downregulated in CML at diagnosis. 

### 3.3. The Upregulation of Both Large HERCs, HERC1 and HERC2, Transcript Levels Are Tightly and Timely Associated with the Disappearance of the Leukemic Cells

BCR-ABL was the first oncogene associated with a neoplasm displaying dual properties, either as a driver or in sustaining the proliferation and expansion of the leukemic clone, facilitating enormously the development of the first selective and specific tyrosine kinase inhibitor (TKI) launched in clinical medicine, Imatinib. Later, the second generation of TKIs (e.g., Nilotinib, Dasatinib) was developed with the aim of counteracting the effect of TKI resistance. Currently, TKIs treatment of CML patients has dramatically improved the prognosis of the disease leading to complete remission in the vast majority of patients [4]. 

Hence, we aimed to investigate the evolution of the *HERC1* and *HERC2* transcript levels in two cohorts of patients treated with two different TKIs, namely Imatinib (*n* = 15) and Dasatinib (*n* = 14), and to assess their trends during the elapsed time between diagnosis and remission.

**Figure 2:**
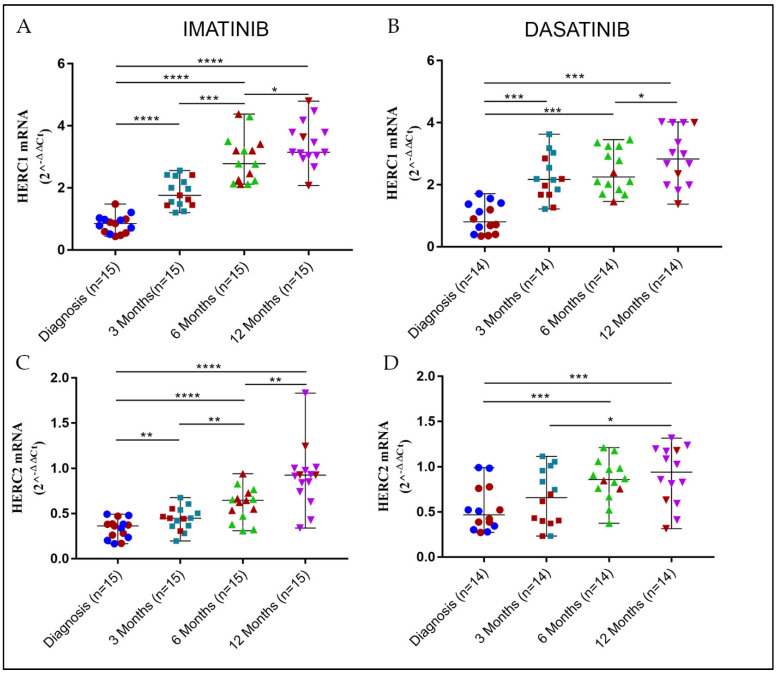
The kinetic evolution of the *HERC1* and *HERC2* transcript levels in CML patients treated with Imatinib and Dasatinib. Independently from the TKIs therapy regimens, both *HERC1* and *HERC2* displayed a transcript accumulation kinetic that was rather comparable. In all cases, the gene expression levels increased over the time elapsed from diagnosis to at least the complete cytogenetic remission (12 months), reaching values comparable to those of healthy subjects. While upon Imatinib treatment, the *HERC1* (**A**) and *HERC2* (**C**) gene transcripts accumulation followed an approximately growing linear trend; with Dasatinib, the *HERC1* (**B**) and *HERC2* (**D**) accumulation differed substantially, being significantly delayed. Red and different color symbols represent BM and PB, respectively, in each time point of CML specimens. *p* ≤ 0.05 = *; *p* ≤ 0.01 = **; *p* ≤ 0.001 = ***; *p* ≤ 0.0001 = ****.

By means of a kinetic at sequential time points from the start of the treatment to complete cytogenetic remission (0, 3, 6, and 12 months), we observed that the gene expression levels of both *HERC1* and *HERC2* significantly changed (Supplementary Table S1). Moreover, to a different extent, the mRNA amount of both genes accumulated over time with slightly different patterns, depending on either the therapy regimens or on the gene of interest (Figure 2). On the contrary, the *BCR-ABL* fusion transcript steeply decreased within the first 3 months from the beginning of the therapy and further smoothly declined until the 12th month (Supplementary Figure S1), indicating that the leukemic clone was disappearing and the normal polyclonal hematopoiesis appeared again.

We also noticed that *HERC1* mRNA amount significantly increased within the first 3 months, with a steeper trend in the case of Dasatinib (median = 2.17) treated patients when compared to the Imatinib counterpart (median= 1.76). Interestingly, at the later time points (6 and 12 months), while in the case of Imatinib, the *HERC1* gene expression displayed a steady increment, with Dasatinib, the increase was still discernible but to a lesser extent (Figure 2A,B). Eventually, in either treatment at the endpoint (12 months), the *HERC1* transcript amounts were comparable (Imatinib median = 3.35; Dasatinib median = 2.83).

Even though at diagnosis, the *HERC2* baseline mRNA level was lower than that of *HERC1*, the gene expression pattern observed was similar. Patients under Imatinib therapy displayed a gradual increase in *HERC2* transcript level and reached values (median = 0.93) comparable to those of the healthy controls at the endpoint (12 months). Unlike *HERC1*, in the case of *HERC2,* the Dasatinib treatment did not lead to a prompt and sharp gene expression increase within the 3 month time window. However, the *HERC2* transcript amount observed after 12 months of Dasatinib therapy was undistinguishable (median = 0.94) from that of the Imatinib counterpart and comparable to those of healthy controls. Noticeably, a significant increase was detected only after 6 months (median = 0.86) from the starting of the Dasatinib treatment (Figure 2C,D). 

Since, in the case of the Dasatinib treated group, genders were not equally represented, we assessed whether the expression of large *HERCs* genes was different in males and females in both groups (Imatinib vs. Dasatinib). Our analysis did not detect any significant differences suggesting that, at least in blood cells, the *HERC1* and *HERC2* gene expression is independent of the gender at diagnosis and during remission (data not shown). 

### 3.4. Inverse and Positive Correlations among Large HERCs and BCR-ABL in CML Patients under TKIs Treatment

**Figure 3:**
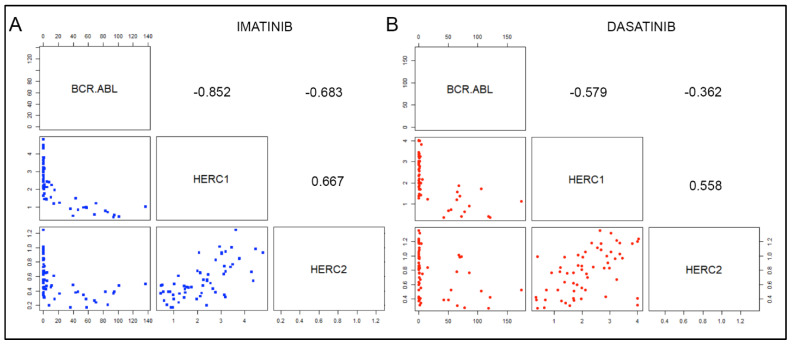
Gene expression correlation analyses among *HERC1*, *HERC2,* and *BCR-ABL* from CML patients treated with two different TKIs. The scatter plots and Spearman coefficient (rho) showed the correlations occurring among the *BCR*-*ABL*, *HERC1,* and *HERC2* transcripts in CML patients’ specimens collected at different time points starting from the beginning of the Imatinib (**A**) and Dasatinib (**B**) treatments up to 12 months (0, 3, 6, and 12). Values are expressed either as 2^−∆∆Ct^ for both *HERC1* and *HERC2* and as I.S. for *BCR*-*ABL*, respectively. Each blue and red solid circle refers to a patient who has been treated either with Imatinib or with Dasatinib, respectively.

The kinetics of the *BCR-ABL*, *HERC1,* and *HERC2* gene expression levels showed potential correlations among the different genes. To sort out the issue, we calculated the Spearman correlation coefficients (*ρ*) amongst the three genes (*BCR-ABL*, *HERC1,* and *HERC2*), either intra- or inter-therapy. In Imatinib-treated patients, we found that both large HERCs negatively correlated with *BCR-ABL* (Figure 3A), while a positive correlation coefficient was calculated between the *HERC1* and *HERC2* (*BCR-ABL* vs. *HERC1 ρ = −*0.85*; BCR-ABL* vs. *HERC2 ρ = −*0.69*; HERC1* vs. *HERC2 ρ =* 0.68).

Conversely, in Dasatinib-treated patients, the correlation coefficients fell sharply, the inverse correlation between *BCR*-*ABL* and *HERC1* being the most significant (*ρ =* −0.57). Though to a lesser extent, we also identified a moderate positive correlation between *HERC1* and *HERC2* (*ρ =* 0.44) (Figure 3B). 

Interestingly, when we attempted to assess the correlation of the large *HERCs* between the two TKIs (i.e., Imatinib and Dasatinib) treated cohorts, we observed a positive correlation for each *HERC* member. However, a better outcome was attained with *HERC1* when compared to *HERC2* (Imatinib *HERC1* vs. Dasatinib *HERC1 ρ =* 0.68; Imatinib *HERC2* vs. Dasatinib *HERC2 ρ =* 0.47).

Furthermore, when we compared the correlation coefficients between Imatinib *HERC1* vs. Dasatinib *HERC2,* and vice versa (i.e., Dasatinib *HERC1* vs. Imatinib *HERC2)*, we found a positive correlation only with the latter pair (Imatinib *HERC1* vs. Dasatinib *HERC2 ρ =* 0.32; Dasatinib *HERC1* vs. Imatinib *HERC2 ρ =* 0.59). 

Overall, these data imply that (a) in CML patients under different TKIs treatment, the gene expression of both large *HERCs* negatively correlate with the *BCR-ABL* transcript; (b) the mRNA amount of the large *HERCs* was equally and significantly downregulated at the onset stage of CML when compared to healthy counterpart; (c) the positive correlation between *HERC1* and *HERC2* in patients treated with Imatinib is comparatively higher (*ρ* = 0.667) than that observed in patients treated with Dasatinib (*ρ* = 0.558). 

### 3.5. The Levels of HERC1 and HERC2 Are Sensitive to BCR-ABL Inhibition

**Figure 4:**
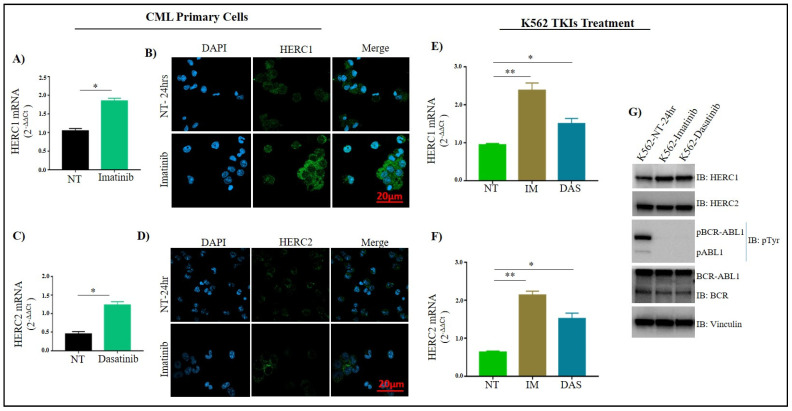
Effect of Imatinib and Dasatinib treatment on HERC1 and HERC2 expression in primary CML and K562 (Ph+) cells. The *HERC1* and *HERC2* gene expressions following Imatinib (1 μM) and Dasatinib (0.25 µM) treatment were determined at mRNA and protein level by RT-qPCR (**A**,**C**,**E**,**F**), immunofluorescence (**B**,**D**), and Western Blotting (**G**). Imatinib (1 µM) and Dasatinib (0.25 µM) treated K-562 cells (24 h) showed an increase in *HERC1* gene expression both at mRNA and protein levels compared to non-treated cells, in contrast, an increase in *HERC2* mRNA was observed without any change in its protein level. TKIs impaired the phosphorylation (enzymatic actively) of BCR-ABL, which indicates the HERC1 protein and mRNA levels of both HERCs are modulated by the activity of Bcr-Abl1. Vinculin was used as a loading control in Western blot. *p* ≤ 0.05 = *; *p* ≤ 0.01 = **.

To assess whether the gene and protein expression of both HERC1 and HERC2 were concurrent, we treated in-vitro for 24 h with Imatinib and Dasatinib, 5 primary BM samples collected at diagnosis, and then evaluated the gene and protein levels. In parallel, the Ph+ K-562 cells were also treated with the same TKIs at the same concentration and for the same time. Interestingly, primary leukemic cells, as well as (Ph+) K-562, showed a significant increase in HERC1 both at mRNA and protein level after TKIs treatment, indicating that by inhibiting the BCR-ABL tyrosine kinase activity, the HERC1 levels were restored. Surprisingly, either in primary CML and K-562 treated cells, we also observed an upregulation of *HERC2* mRNA level that was not concurrent with the respective protein level (Figure 4). We also determined whether there was a time dependency in the accumulation of the *HERC1* and *HERC2* transcripts amounts in K-562 cells upon TKIs treatments. Indeed, we observed that, over a 24 h time window, the accumulation of *HERC1* and *HERC2* displayed a pretty similar kinetic, being faster in the case of Dasatinib (Supplementary Figure S3).

### 3.6. Leukemic Cells Vary HERC1 Levels upon Differentiation Induction

Previous findings indicate that upon Imatinib treatment, and prior to apoptosis, CML cells undergo erythroid differentiation [26]. This, alongside our current data, prompted us to investigate whether *HERC1* and *HERC2* cellular levels were associated with differentiation stages. Hence, we determined the HERC1 protein amount in different blood cell types in both healthy donors and CML patients. We observed that HERC1 is mainly downregulated in the myeloid lineage, as well as early precursors, from CML primary cells when compared to the healthy counterparts (Figure S2). 

Based on these preliminary results, we suspected that different HERC1 levels might be associated with diverse blood cell types. Therefore, we attempted to differentiate K-562 cells with Erythropoietin (EPO) and phorbol 12 myristate 13-acetate (PMA). It is known that erythropoietin induces K-562 cells toward the erythroid-like lineage [23], while PMA toward megakaryocytic-like cells [24].

**Figure 5:**
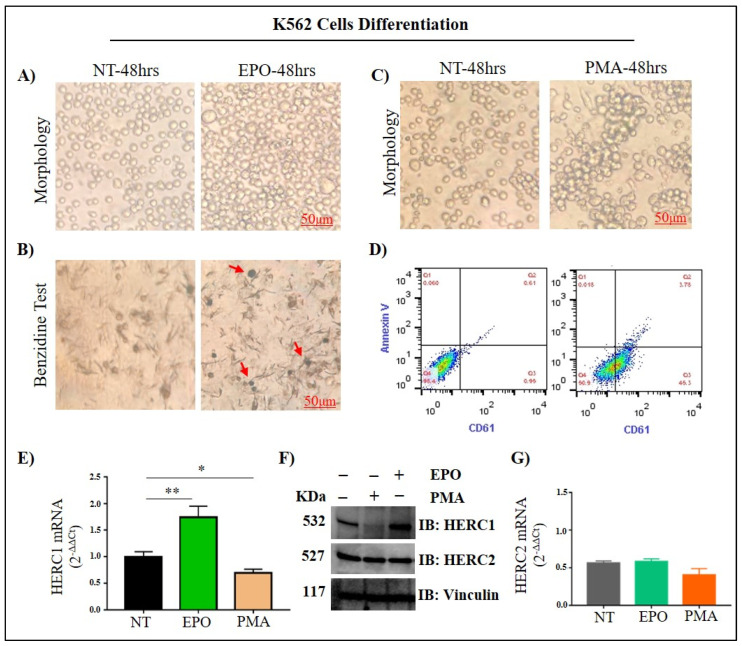
Leukemic cells’ differentiation towards the erythroid and megakaryocyte-like lineages antagonistically affect the HERC1 levels. Differentiation was performed by incubating Ph+ K562 myeloid leukemia cell lines with 250 nM phorbol 12 myristate 13-acetate (PMA) and 20 U/mL Erythropoietin (EPO). (**A**) Representative pictures of the morphology of uninduced control and differentiated cells; (**B**) K562 cells were harvested after 48 h with or without EPO induction and stained with benzidine. Benzidine-positive cells turned blue, indicated with red arrows. Untreated K562 cells served as a control. (**C**,**D**) Representative cell morphology images and flow cytometry analysis of PMA treated K562 cells. Prior flow cytometry cells were stained for megakaryocyte-specific cell surface marker CD61. (**E**,**F**) Expression changes in *HERC1* gene at mRNA and protein level in non-treated, EPO, and PMA induced K562 cells. An increase in HERC1 protein and mRNA level was seen in EPO-treated K562 cells, while a decrease in HERC1 gene expression was observed in PMA treated cells. (**G**) Interestingly, there is not any change in HERC2 protein level in K562 differentiated cells. *p* ≤ 0.05 = *; *p* ≤ 0.01 = **.

After EPO treatment, and when compared to the untreated control, K562 cells differentiated into enlarged erythroid-like cells, as was confirmed by benzidine assay. EPO induced an increase of erythroid differentiation by approximately 35% (benzidine-positive cells) with respect to control (Figure 5A,B). 

Conversely, PMA treated K-562 cells tend to differentiate towards the megakaryocyte lineage with elongated shapes and acquired cell-to-substratum adhesion properties. PMA-induced megakaryocytic differentiation was confirmed by a significant increase in megakaryocyte-specific cell surface marker CD61 (Figure 5C,D). After differentiation, total RNAs and proteins were extracted, and *HERC1* mRNA and protein expression was analyzed by RT-qPCR and Western Blot. Interestingly, HERC1 levels both at mRNA and protein were upregulated when cells were induced to differentiate towards the erythroid lineage after EPO treatment. On the contrary, when cells were induced towards the megakaryocyte lineage, the mRNA and protein amount were greatly reduced (Figure 5E,F). On the contrary, we did not detect any significant change in terms of HERC2 mRNA and protein levels (Figure 5F,G).

To further corroborate these data and exclude that our observations might be cell-specific, we also attempted to differentiate a Ph negative cell line, Human Erythroleukemia (HEL), toward megakaryocytic-like cells, by using phorbol 12-myristate 13-acetate (PMA). After PMA incubation, many cells underwent morphologic changes becoming elongated and firmly attached to the substratum (Supplementary Figure S4A), alongside an increased expression of the CD61 cell-specific surface marker (Supplementary Figure S4B,C). Similar to what we observed in Ph+ cells, the *HERC1* expression, both at mRNA and protein level, was downregulated upon differentiation. By contrast, the transcript and protein amounts of HERC2 remained unchanged and comparable to those of the non-treated cells (Figure S4D–F). Overall, these results suggest that HERC1 might be potentially implied in the remodeling of the cellular proteome during blood cell differentiation and thus represents a novel differentiation marker. 

## 4. Discussion

The dysregulation of the Ubiquitin-Proteasome System (UPS) has been reported to substantially contribute to the initiation and progression of CML as well as maintenance of the Leukemic Initiating Cell (LIC) [27]. Furthermore, the anomalous UPS regulation is also implied during the progression from chronic to accelerated and blast crises [28]. In addition, in acute lymphoblastic leukemia, somatic deletions of *HERC1* were associated with lower levels of the protein MSH2, which is in turn involved in DNA mismatch repair [29]*,* and mutations in *HERC1* were also found in T-ALL patients [30]. Recurrent mutations of both *HERC1* and *HERC2* have also been detected in T cell prolymphocytic leukemia with a frequency of approximately 10% [31]. Previously, and in more detail than the present study, we observed a tight and unambiguous inverse correlation between the presence of the fusion oncogene *BCR-ABL* and the gene expression of *HERC1* and *HERC2* in newly diagnosed CML patients. It is noteworthy that when compared to healthy donors, in CML specimens, the *HERC1* and *HERC2* mRNA amounts are detected at lower levels in white blood cells precursors, as well as in mature white blood cells, as determined in BM and PB specimens. We found that in CML cells the gene and protein expression of HERC1 seem to be sensitive to TKIs, whereas in the case of HERC2 such behavior was restricted only to the transcript. Eventually, for the first time our data display that in healthy mature blood cells there is a greater accumulation of large *HERCs* and that, in terms of absolute values, the large *HERCs* transcript amount profoundly differs being *HERC2* expressed at a remarkably lower extent when compared to *HERC1.* Overall, these findings let surmise a putative role of both giant HERCs in the chronic phase of the CML. 

Currently, the mechanistic and molecular reasons by which the genes encoding for the large HERCs are downregulated in CML are unknown. In spite of that, CML displays a peculiar *BCR-ABL* dependent gene signature conferring unique leukemogenic properties to the cells, including the aberrant readout of coordinated effects of transcription factors as well as aberrant epigenetic controls. Additionally, other post-transcriptional mechanisms, including non-coding RNA-mediated specific mRNA silencing, are also considered to be very powerful epigenetic mediators modulating the CML expression profiles and phenotypic outcome [32]. Overall, it is likely that BCR-ABL exerts a negative effect on the accumulation of these *HERC* genes. Recently, we observed that BCR-ABL interacts with HERC1 and that the latter is a substrate of the former [20]. Determining the molecular mechanism/s by which large *HERCs* are downregulated in CML and ascertaining to what levels the aberrant *HERCs* transcripts dysregulation occurs will be a matter of future studies.

Interestingly, our findings point out that the two large *HERCs*, *HERC1* and *HERC2*, genes are differently controlled by tyrosine kinases. Alongside BCR-ABL, Src kinases play a role in the leukemogenesis of CML. The Src kinases sustain the proliferation of BCR-ABL expressing cells, the transition of CML to lymphoid blast crisis [33,34,35], and in some cases, the overexpression of Src kinases have been implicated in Imatinib acquired resistance [7,8]. Accordingly, activation of Src may promote BCR-ABL phosphorylation within the activation loop, thus interfering with the Imatinib binding. Therefore, the small molecule inhibitor with overlapping activity against both Abl and Src (i.e., Dasatinib) results in enhanced activity against CML compared with that of Imatinib, which is a potent Abl tyrosine kinase inhibitor but has negligible activity against Src kinases [10,36]. Our current evidence suggests that, though *HERC1* and *HERC2* are localized on the same chromosome [37] and encode for proteins sharing many structural similarities, their gene expression might be differentially regulated by Src family kinases. Indeed, though both genes appear to be severely downregulated in chronic myeloid leukemia cells, the effect of Dasatinib on the *HERC1* and *HERC2* gene expression is different. As a matter of fact, when compared with *HERC1*, *HERC2* appeared more sensitive to the effects of Dasatinib with a slower recovery kinetic. These data prompt us to speculate that the transcription of both genes is negatively regulated by BCR-ABL. Additionally, our results suggest that at least in the early stages of recovery, *HERC2* gene expression might be under positive regulation by Src kinases. We suppose that, differently from *HERC1*, the *HERC2* gene expression is under a triple control depending on the chromatin condensation grade, which represents a hindrance for the transcription factors’ accessibility to the promoter. In support of this assumption, an in-silico survey (http://gene-regulation.com/pub/programs/alibaba2/) revealed that a conserved DNA consensus binding site for a transcription factor downstream of Src, Myc, exists exclusively on the *HERC2* gene promoter, but not on that of *HERC1*.

What are the biological consequences of keeping large *HERCs* transcript amount low during myeloid neoplastic proliferation? At the moment, it remains an open question, though some plausible working hypotheses should be carefully considered. Among the different neoplasm, the CML is characterized by a very high genomic instability due to alterations of the DNA damage responses and repair as well as cell-cycle checkpoints. Overall, these alterations lead to the acquisition of secondary genetic lesions that accompany the progression from chronic to accelerated and eventually blast crisis [38,39]. The loss of p53 function has been associated with the suppression of apoptosis and progression of CML into blast crisis [40]. In this respect, HERC2 also could provide an important point of regulatory flexibility, allowing cells to finely control their response to specific genotoxic insults and maintain genome stability by controlling the XPA and BRCA1 protein stability and eventually regulating the cell-cycle at G2/M checkpoint transition [41,42,43]. Moreover, HERC2 has been shown to physically interact, via the CPH domain, with the oncosuppressor protein p53. Moreover, by finely tuning p53 tetramerization HERC2 controls the p53 DNA binding and, thus, its transcriptional activity [44]. Among the outstanding hallmarks of neoplastic cells, there is the acquired ability of the cells to escape the programmed cell death, a process that is strictly under the control of the ubiquitin-proteasome system [45,46], where E3 ligases are crucial players regulating many aspects of the process. Though HECT family members are a minority of the whole E3 ubiquitin ligases, few of them appear to be pivotal either in enhancing (i.e., NedL1) or inhibiting (i.e., AREL1) p53 mediated apoptosis [47,48]. It has been recently reported that HERC5 regulates the apoptosis rate in colorectal cells by acting throughout CtBP1, and thus regulating the expression of pivotal apoptotic genes (e.g., BAX, PUMA, BIK) [49]. Our findings do not allow us to discern to what degree HERC1 and HERC2 are involved in regulating apoptosis; hence this would be one of the next challenging issue to be sorted-out. 

A common and prominent feature shared by all leukemia, as well as many other tumors, including solid cancers, is that they display impaired cell differentiation. Basically, the differentiation defects featuring leukemic cells, though to a different extent, are common to every leukemia form, including the myeloid-related disorder. Whereas the functional role of HERC2 has been extensively investigated, that of HERC1 has only been recently associated with cellular proliferation. However, with the exception of a few observations, restricted to the central nervous system in which HERC1 seems to be important for the axonal myelination and the control of the presynaptic membrane dynamics of central synapses [50], the role of this giant ubiquitin-ligase during cell-differentiation remains poorly investigated. Nonetheless, it is well known that the UPS plays an important role during morphogenesis and differentiation in almost every cell ranging from lower eukaryotes [51,52,53,54,55,56,57] to mammals [58,59], including blood cells [60]. In this regard, red blood cells differentiation requires the remodeling of their proteome that takes place by the selective degradation of pre-existing proteins through the UPS.

Our findings indicate that HERC1 protein amount is associated with the differentiation process of leukemic cells at least towards two different lineages, suggesting that HERC1, and to a lesser extent HERC2, might contribute to the proteome remodeling associated with cell differentiation. Furthermore, the endpoint of cellular differentiation is cell senescence, a process characterized by a stable cell cycle arrest and where autophagy plays a critical role [61]. At this point, it remains to be ascertained at which point large HERCs might be crucial contributors to all these processes. Further research work is required to investigate the functional role of HERC1 in the regulation of blood cell differentiation and identify what the HERC1 gene expression regulators are.

## 5. Conclusions

Our survey reveals that the *HERC2* gene is expressed in PB and BM from healthy subjects with lower levels in the latter specimens. In white blood cells, the *HERC2* transcript amount is inferior to that of *HERC1*. As it occurs for *HERC1*, also the *HERC2* gene expression is profoundly reduced in CML patients at diagnosis, but it is restored to normal levels and with a relatively rapid kinetic upon disease remission. Eventually, our evidence suggests that, differently from *HERC1*, the *HERC2* transcription is likely under the control of Src family kinases. We also observed that the HERC1 mRNA and protein amount levels are tightly linked to the cell fate. Remarkably, our findings suggest that, at least in blood cells, HERC1 might be a novel differentiation marker.

## Figures and Tables

**Table 1:** Baseline characteristics of healthy subjects and CML patients.

Groups	TKI Therapy Dosage (mg/d)	No. of Samples	Age		Gender		BCR-ABL (IS) % at Onset		Rearrangement			Mutations
			Range	Median	M	F	Range	Median	p190	p210	p230	Ex8/Int8
Control	/	16	21–63	36	9	7	/	/	/	/	/	/
Imatinib	400	15	37–83	54	8	7	36.5–137.0	69.50	/	15 ^#^	/	2
Dasatinib	100	14	21–72	63.5	10	4	42.0–174.0	72.00	/	14 ^##^	/	1

^#^ 2 out of 15 patients have coexistence of p210(b3a2) and p210(b2a2), ^##^ 1 out of 14 patients have coexistence of p210(b3a2) and p210(b2a2). M = Male, F = Female.

## Data Availability

We did not produce a public database.

## Supplementary Materials

The following supporting information can be downloaded at: https://www.mdpi.com/article/10.3390/jcm11020324/s1, Figure S1. Anticorellation between large *HERCs* and *BCR-ABL* transcript amounts in CML patients. Figure S2. HERC1 is sharply downregulated in the leukemic myeloid lineage. Figure S3. Time dependency effect of TKIs (Imatinib and Dasatinib) on HERC1 and HERC2 transcript level in K562 (Ph+) cells. Figure S4. (A) Representative images of K562 cell pellet showing reddish color and blue coloration in benzamine test due to the presence of hemoglobin after EPO treatment (B) immunofluorescence microscopy revealing the EPO-induced differentiation of K562 cells resulted in an increase of HERC1 protein levels. (C,D) Representative cell morphology images and flow cytometry analysis of HEL cells stained using anti-CD61 megakaryocyte specific cell surface marker of undifferentiated and differentiated HEL-cells, after 48hours in the presence of PMA (250 nM). (E,F) *HERC1gene* expression both at mRNA and protein in undifferentiated and differentiated HEL cells. The a-tubulin and vinculin were used as loading controls in Western Blots. Table S1. *p*-values of *HERC1* and *HERC2* by time in CML patients under different TKIs treatment by Kruskal-Wallis test.
