# Supplementary material for: The Downregulation of Both Giant HERCs, HERC1 and HERC2, Is an Unambiguous Feature of Chronic Myeloid Leukemia, and HERC1 Levels Are Associated with Leukemic Cell Differentiation
Source: J Clin Med. 2022 Jan 10;11(2):324. doi: 10.3390/jcm11020324 (PMC8778248; doi:10.3390/jcm11020324)
Supplement: Supplementary file 1 [file jcm-11-00324-s001.zip › jcm-1517224-SI.pdf]

Supplementary Figures

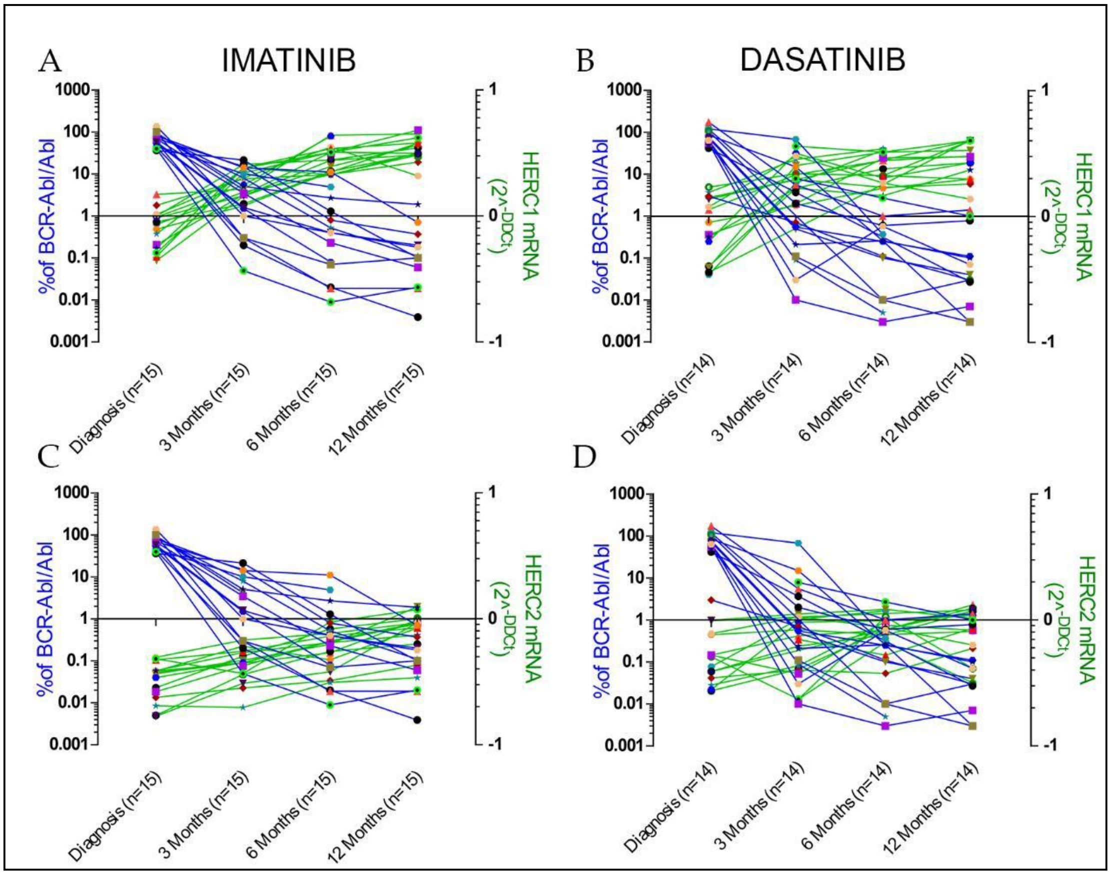

Figure S1. Anticorellation between large *HERCs* and *BCR-ABL* transcript amounts in CML patients. An inverse correlation between large *HERCs* and *BCR-ABL* gene expression was observed at different time points (diagnosis, 3, 6 and 12 months) of disease under tyrosine kinase inhibitor Imatinib (**A** & **C**) as well as Dasatinib (**B** & **D**) treatment in CML patients. There is a rapid downregulation of BCR-ABL in patients treated with Dasatinib in the first three months of treatment with a median value of 0.65 when compared to a downregulation with Imatinib (median = 3.5). This pattern of sequential down-modulation of BCRABL1 indicating that might be with the eradication of sick cells/clone (Ph+) and re-appearance of normal cells/polyclonal hematopoiesis the expression of large *HERCs* upregulate gradually. A base-10 log scale is used for the both right and left Y-axis.

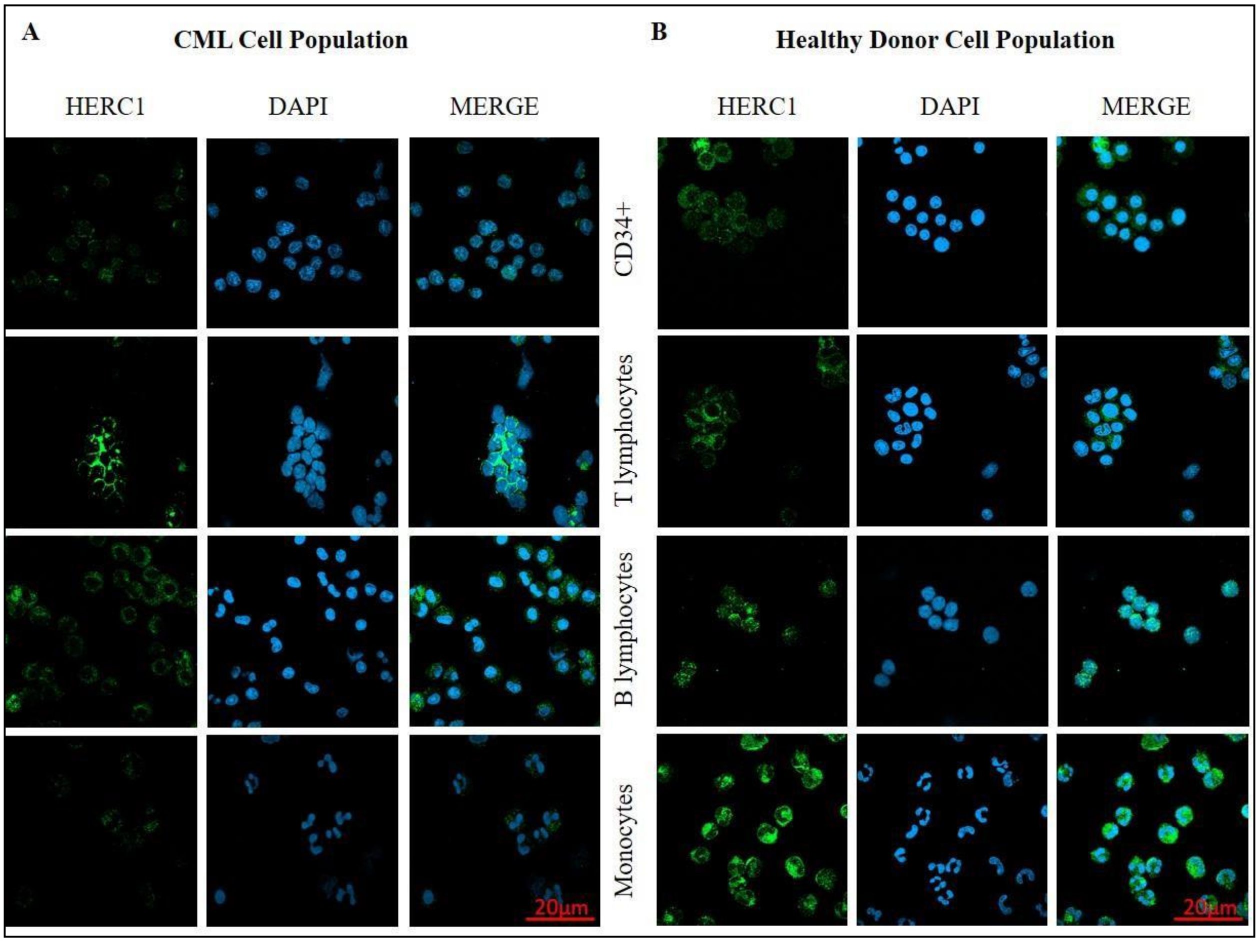

Figure S2. HERC1 is sharply downregulated in the leukemic myeloid lineage. Immunofluorescence staining assay was performed on different cell population after magnetic isolation from a healthy donor and of CML bone marrow (BM) samples. The green signal corresponds to HERC1 while blue DAPI is used to stain nuclei. (**A**) HERC1 protein expression in different cells separated from CML BM specimen. (**B**) HERC1 protein expression in healthy donor cell population. HERC1 protein expression is relatively low in CD34+ and mononuclear cells (mainly Myeloid cells population) derived from CML BM specimen compared with corresponding cell population of the healthy donor control. While the HERC1 protein level in T and B- lymphocytes is comparable in sick and healthy cell population and it displays an intense and cytosolic localization.

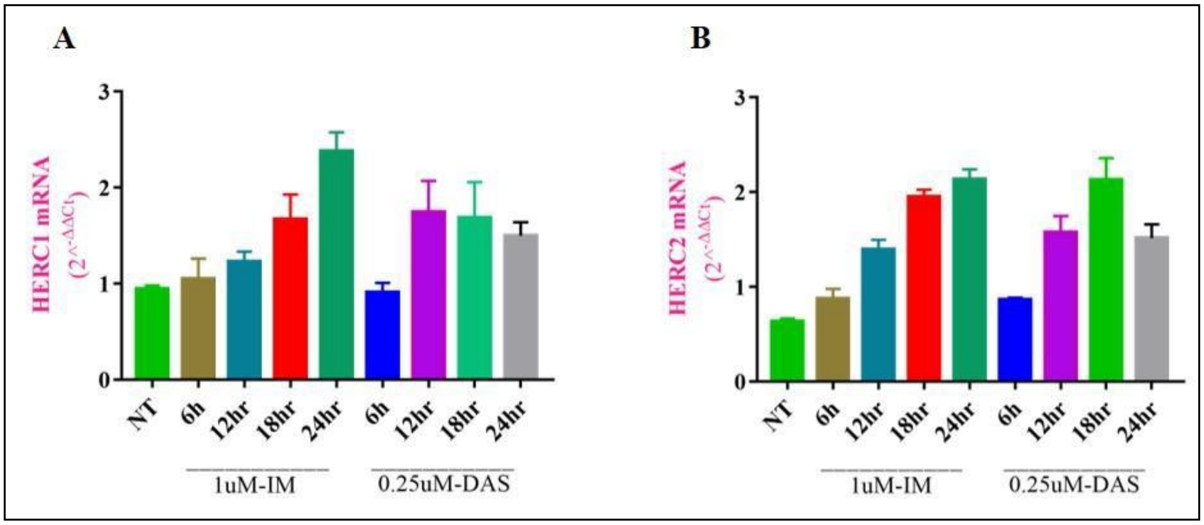

Figure S3. Time dependency effect of TKIs (Imatinib and Dasatinib) on HERC1 and HERC2 transcript level in K562 (Ph+) cells. The *HERC1* (**A**) and *HERC2* (**B**) gene expression following Imatinib (1µM) and Dasatinib (0.25 µM) treatment at different time points were determined at mRNA level by RT-qPCR. Imatinib and Dasatinib treated K-562 cells showed an increase in *HERC1* and *HERC2* mRNA compared to non-treated cells in a time- and TKI-dependent way.

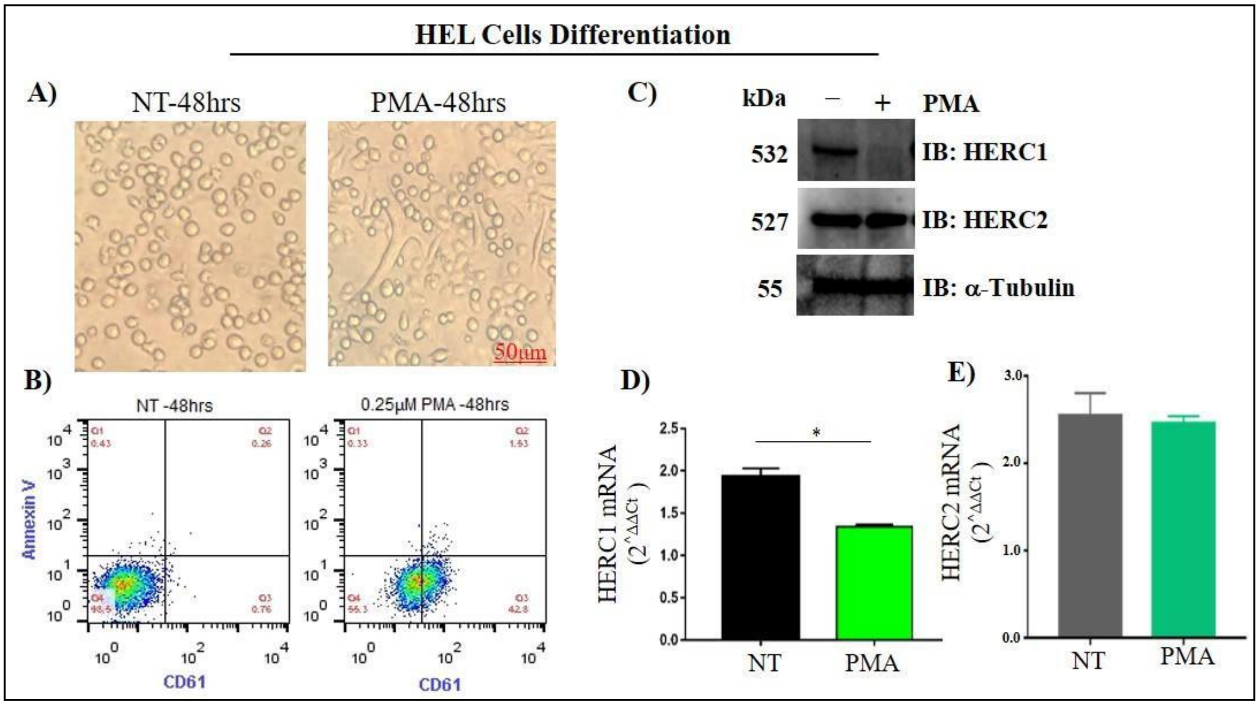

Figure S4. (**A**) Representative images of K562 cell pellet showing reddish color and blue coloration in benzamine test due to the presence of hemoglobin after EPO treatment B) immunofluorescence microscopy revealing the EPO-induced differentiation of K562 cells resulted in an increase of HERC1 protein levels. (**C & D**) Representative cell morphology images and flow cytometry analysis of HEL cells stained using anti-CD61 megakaryocyte specific cell surface marker of undifferentiated and differentiated HEL-cells, after 48hours in the presence of PMA (250nM). (**E & F**) *HERC1*gene expression both at mRNA and protein in undifferentiated and differentiated HEL cells. The a-tubulin and vinculin were used as loading controls in Western Blots.

Table S1: p-values of *HERC1* and *HERC2* by time in CML patients under different TKIs treatment by Kruskal-Wallis test

Gene	IMATINIB	DASATINIB
HERC1	5.597e-10	1.798e-6
HERC2	8.259e-7	0.005179
